# DEPRESSIVE SYMPTOMS, NOT FUNCTIONAL STATUS, PREDICT MORBIDITY AND MORTALITY IN OLDER ADULTS WITH HEART FAILURE

**DOI:** 10.1093/geroni/igae098.1445

**Published:** 2024-12-31

**Authors:** Abigail Latimer, Jia-Rong Wu, Christopher Knoepke, JungHee Kang, Debra Moser

**Affiliations:** University of Kentucky, Lexington, Kentucky, United States; University of Kentucky, Lexington, Kentucky, United States; University of Colorado Anschutz Medical Campus, Aurora, Colorado, United States; University of Kentucky, Lexington, Kentucky, United States; University of Kentucky, Lexington, Kentucky, United States

## Abstract

**Background:**

Older adults (≥ 65 years) (OAs) with heart failure (HF) are at high risk of functional impairment and co-morbid depression. The impact on morbidity and mortality of these comorbidities in OAs with HF has not been well-studied. OBJECTIVE: To determine the extent to which functional status and depression predicted event-free survival (i.e., hospitalization or death) in OAs with HF.

**Methods:**

We performed a secondary analysis from the Research Interventions in Cardiovascular Health HF Database of OAs with data on depressive symptoms using the Patient Health Questionnaire- 9 (PHQ-9) and functional status using the Duke Activity Status Index (DASI). Patients were followed for 2- years. Event-free survival was determined using Cox proportional hazards models.

**Results:**

We included 157 OAs (mean age= 73 ± 6, 68.2% men, 87.9% White, 63.1% NYHA III or IV). Participants had an average DASI score of 11.49 ± 9.26 and 58% had at least mild depressive symptoms. At time of follow- up, 63 hospitalizations and 25 deaths occurred. The risk of hospitalization or death was 2.16 times greater for OAs who had depressive symptoms (95% Confidence Interval=1.27- 3.67, p <.01) compared to those without, regardless of functional status. The overall model showed a significant improvement in fit relative to the null [x2 (3)= 11.21, p <.05].

**Conclusion:**

Caring for OAs with HF requires attending to depressive symptoms, given the independent risk of hospitalization and mortality, even in the company of varying functional status. Future research should evaluate effectiveness of depression treatments for these patients.

